# An integrative strategy for quantitative analysis of the N-glycoproteome in complex biological samples

**DOI:** 10.1186/1477-5956-12-4

**Published:** 2014-01-15

**Authors:** Ji Wang, Chuang Zhou, Wei Zhang, Jun Yao, Haojie Lu, Qiongzhu Dong, Haijun Zhou, Lunxiu Qin

**Affiliations:** 1 Department of Surgery, Huashan Hospital, Fudan University, Shanghai 200040, China; 2 Institutes of Biomedical Sciences, Fudan University, Shanghai 200032, China; 3 Affiliated Hospital of Xuzhou Medical College, Xuzhou, Jiangsu 221002, China

**Keywords:** ^18^O labeling, Hepatocellular carcinoma, Mass spectrometry, N-glycoproteome

## Abstract

**Background:**

The complexity of protein glycosylation makes it difficult to characterize glycosylation patterns on a proteomic scale. In this study, we developed an integrated strategy for comparatively analyzing N-glycosylation/glycoproteins quantitatively from complex biological samples in a high-throughput manner. This strategy entailed separating and enriching glycopeptides/glycoproteins using lectin affinity chromatography, and then tandem labeling them with ^18^O/^16^O to generate a mass shift of 6 Da between the paired glycopeptides, and finally analyzing them with liquid chromatography-mass spectrometry (LC-MS) and the automatic quantitative method we developed based on Mascot Distiller.

**Results:**

The accuracy and repeatability of this strategy were first verified using standard glycoproteins; linearity was maintained within a range of 1:10–10:1. The peptide concentration ratios obtained by the self-build quantitative method were similar to both the manually calculated and theoretical values, with a standard deviation (SD) of 0.023–0.186 for glycopeptides. The feasibility of the strategy was further confirmed with serum from hepatocellular carcinoma (HCC) patients and healthy individuals; the expression of 44 glycopeptides and 30 glycoproteins were significantly different between HCC patient and control serum.

**Conclusions:**

This strategy is accurate, repeatable, and efficient, and may be a useful tool for identification of disease-related N-glycosylation/glycoprotein changes.

## Background

The glycosylation of proteins is a common post-translational modification. The occupancy of the glycosylation site and the glycan structure in the glycoproteins have a profound effect on their biological functions [1]. Alteration of this glycosylation influences growth, differentiation, transformation, adhesion, metastasis, and immune surveillance of cancers [2-6]. Glycans are classified as either O-linked (through Ser or Thr) or N-linked (through Asn on the Asn-X-Thr/Ser recognition sequence, X ≠ P) depending on their polypeptide attachment site. In particular, N-linked glycosylation is prevalent in secreted proteins found in body fluids (such as blood and urine) [7] and plays a significant role in cellular recognition and signal transduction and can therefore be considered a potential therapeutic target or biomarker for diseases, including cancers [8,9].

Currently, the most effective and accurate method of quantitatively analyzing glycopeptides and glycoproteins is mass spectrometry (MS). MS is usually combined with other techniques such as various protein/peptide enrichment, labeling, and data analysis techniques to obtain a complete understanding of protein glycosylation patterns (including glycosylation sites), site occupancy, and glycan structures. However, accurate, quantitative, high-throughput techniques for comprehensive analyses of protein glycosylation in complex biological samples have only rarely been established [8,10].

N-glycosylated sites in a glycopeptide are usually labeled and identified with ^18^O. Kuster et al. reported a method in which N-linked glycans were enzymatically removed from glycopeptides by peptide N-glycosidase F (PNGase-F), and the glycosylated Asn residues labeled with ^18^O. They demonstrated that the process generated a mass shift of 2 Da and the glycosylated sites were subsequently identified accurately via MS [11]. Kaji et al. further modified this method, performing quantitative comparative analyses using ^16^O-labeled residues as a control. However, the partial overlap in isotopic distribution of the ^16^O- and ^18^O-labeled peptides affects the accuracy of this quantitative method [12]. Recently, Liu et al. established the tandem ^18^O stable isotope labeling technique, which includes enriching glycopeptides via hydrophilic affinity extraction and labeling three ^18^O or ^16^O tandems at their C-terminus and N-glycosylation sites. The mass shift between paired ^18^O- and ^16^O-labeled glycopeptides is 6 Da [13]. This method overcomes isotope distribution overlap and enhances the accuracy of quantification. However, these ^18^O labeling-related techniques are time-consuming and low-throughput due to a lack of software for automatic quantitative analysis [13], particularly when analyzing a large number of complex biological samples. Moreover, these methods are not able to identify glycan structure alterations in specific glycoproteins, which are important for understanding the effects of glycan changes in glycoproteins on pathological processes.

Lectin affinity chromatography is an accurate glycan separation technology, and is extensively used to expound upon the glycan structure in glycoproteins [14,15]. For example, using this technique, *Lens culinaris* (LCH)-affinitive alpha-fetoprotein (AFP-L3) is separated, which is more accurate in diagnosing liver cancer than total AFP [16-19]. In the present study, we combined lectin affinity chromatography, tandem ^18^O/^16^O labeling, MS, and a self-build automatic quantitative method based on Mascot Distiller software to develop an integrated strategy for high-throughput quantitative analysis of N-glycosylation changes in complex biological samples. The accuracy and repeatability of this strategy were verified using glycoprotein standards. We also utilized this strategy to analyze the serum of healthy individuals and hepatocellular carcinoma (HCC) patients to confirm its feasibility in complex biological samples.

## Results and discussion

### An integrated strategy for glycoproteomic study

It is well known that protein glycosylation varies widely between different glycoproteins; indeed, a single glycoprotein can be glycosylated at multiple sites with various glycans. Such complexity makes it difficult to characterize protein glycosylation patterns on a proteomic scale. The major limitations of the current glycoproteomic studies include: (1) Searching for changes in glycan structure or glycosylation site occupancy of a single glycoprotein, rather than in a high-throughput manner on a large proteomic scale [16,20]; (2) Investigating glycosyltransferase or the general changing trends of glycan structure in biological samples, regardless of the glycan structure of each specific glycoprotein [21-23]; and (3) Analyzing the expression levels of glycoproteins with specific glycan structure, but not describing the glycosylation sites and glycosylation site occupancy [24-27]. In order to overcome these limitations, we developed an integrated strategy which can be used to quantitatively analyze the abundance of glycoproteins/glycopeptides in a high-throughput manner, as well as the glycan structure and sites of altered N-glycosylation.

**Figure 1:**
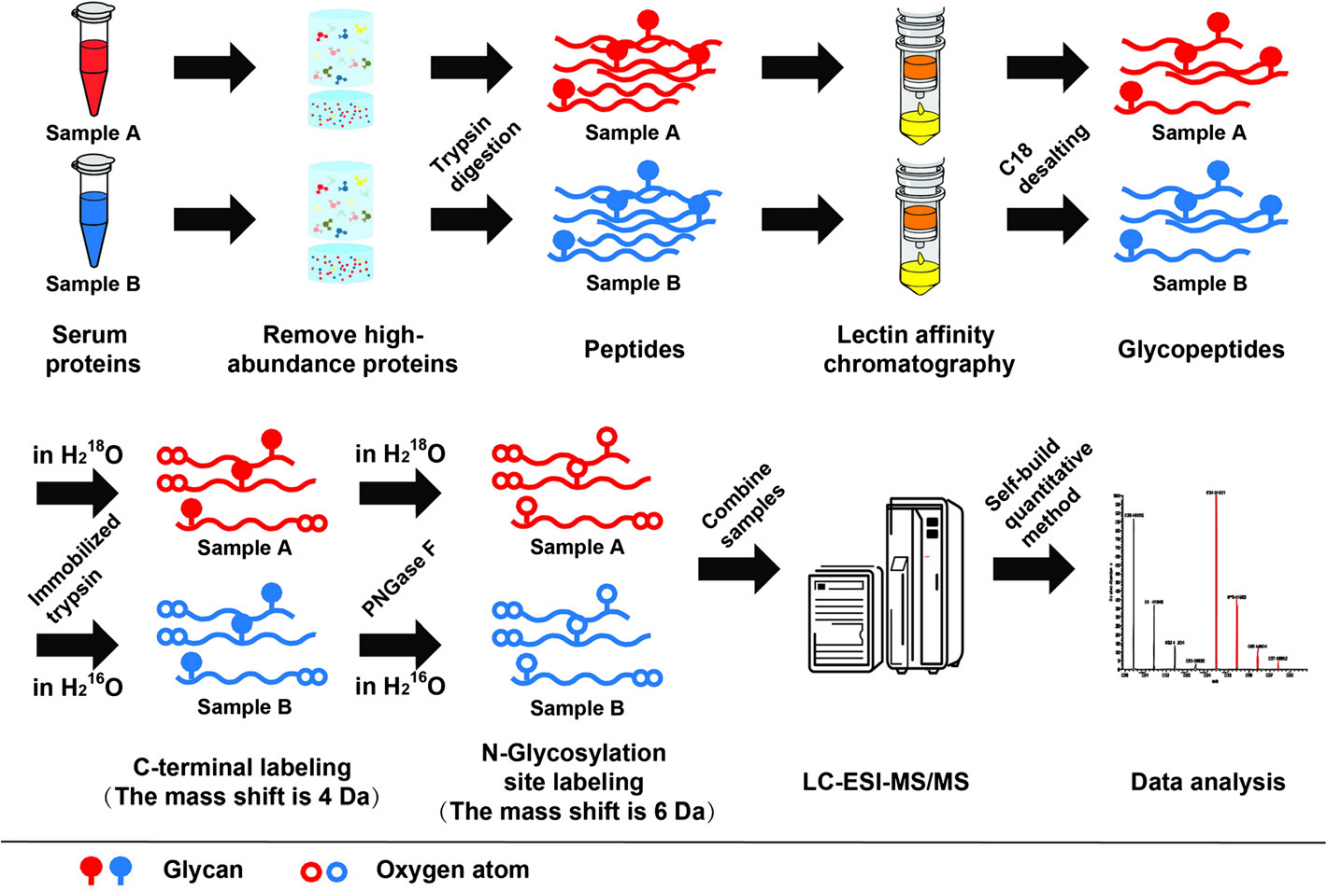
**Flow chart of the integrated strategy.** Serum samples were digested with trypsin after removal of high-abundance proteins. Glycopeptides were enriched with lectin chromatography from two different samples, desalted, and then catalyzed by immobilized trypsin and PNGase-F in ^18^O or ^16^O water, as indicated. Equal amounts of ^18^O- and ^16^O-labeled glycopeptides were mixed, and the 6 Da mass shifts were generated between paired, labeled glycopeptides, which could be identified by subsequent LC-ESI-MS/MS. The data were quantitatively analyzed automatically with the self-build quantitative method.

Our overall experimental strategy is shown in Figure 1. The glycopeptides with specific glycan structure were enriched via digestion and lectin affinity chromatography, and then treated with immobilized trypsin and PNGase-F in ^16^O or ^18^O water. During this process, the C terminus of the peptide was labeled by two ^18^O or ^16^O, and the N-glycosylation site was labeled by one ^18^O or ^16^O. Thus, when they were mixed at the same ratio, a mass shift of 6 Da was generated between the paired ^18^O- and ^16^O-labeled glycopeptides, while only a mass shift of 4 Da was present between the paired non-glycopeptides, which could subsequently be identified by MS. A detailed description of ^18^O labeling is supplied in Additional file 1. The relative concentration ratio could be directly quantitated through the relative signal strength of the peptide ion pair in the precursor scan because corrections for the overlapping distributions of monoisotopic peaks were built into the software.

The glycan structure of glycoproteins is specifically recognized by lectin and the glycan structure changes of glycoproteins distinguished by LCH, WGA, or ConA are associated with cancer development, and therefore have potential diagnostic and prognostic values [28-31]. As such, in this study, we used LCH, WGA, and ConA lectin chromatography to separate and enrich glycopeptides with a specific glycan structure. Using this method, we were able to obtain information regarding changes in the abundance of glycoproteins with different types of glycan, as well as the glycosylation site and glycosylation site occupancy in each altered glycoprotein, all with one experiment. Our strategy pinpoints the glycosylation changes to each glycoprotein on a large glycoproteomics scale, providing a valuable supplement to techniques currently used in glycoproteomics.

However, lectin affinity chromatography is far from ideal [32-34], as the enrichment efficiency of the method is unsatisfactory, is easily affected by buffer conditions, and non-specifically recognizes glycans [35,36]. In this study, we attempted to stabilize the binding and elution buffers, including adjustment of pH, concentration, and binding/eluting time, in different experiments, to overcome these disadvantages.

Incomplete ^18^O labeling generates negative results, primarily due to the reversible labeling reaction at the C-terminus [37]. A number of factors may affect the efficiency of ^18^O labeling at the C-terminus, such as the catalytic activity of trypsin, the purity of H_2_^18^O, H_2_^16^O, and other reagents, the back-exchange caused by incomplete trypsin quenching, and the relative positions of Lys and Asp/Glu at the C-terminus [38-40]. In order to remove these interference factors, we made the following modifications to our experiments based on previous studies: (1) Immobilized trypsin was applied to increase the mole ratio of protease to substrate and improve labeling efficiency [41]. The immobilized enzymes could be completely removed physically after the reaction and the carboxyl oxygen exchange nearly ceased and reduced back-exchange; (2) Acidic conditions were adopted to facilitate catalysis of the carboxyl oxygen reaction and obtain better efficiency of the immobilized trypsin labeling [40,42]; and (3) After digestion with trypsin, samples were boiled for 10 min followed by freezing for 5 min, and methanoic acid was added prior to PNGase-F labeling to fully quench the trypsin and avoid back-exchange [39].

### Validation of the feasibility and accuracy of the integrative strategy

**Figure 2:**
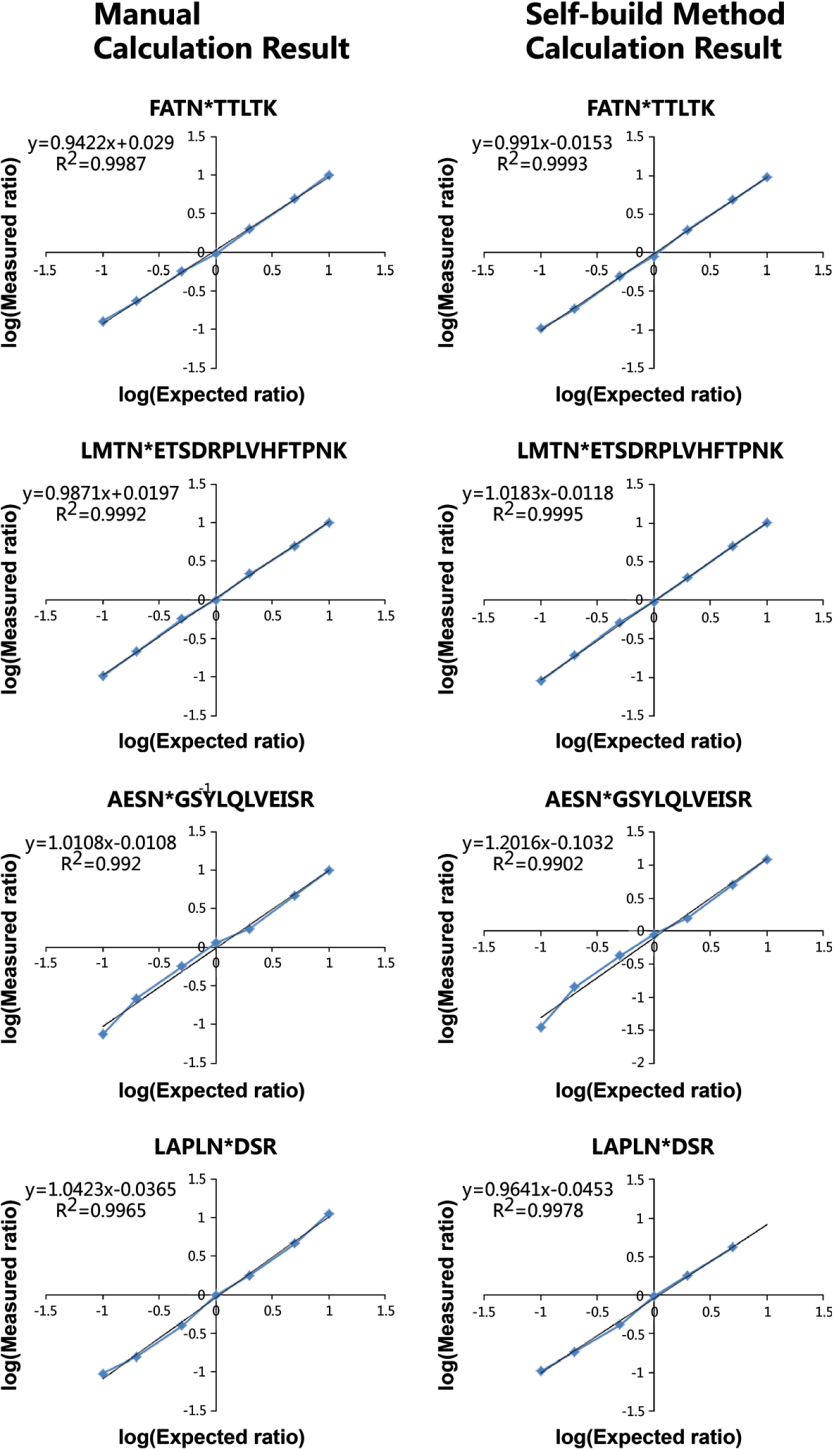
**Linearity and accuracy of the self-build quantitative method in glycopeptides from glycoprotein standards.** Four glycopeptides, FATN*TTLTK and LMTN*ETSDRPLVHFTPNK from invertase [Swiss-Prot:P00724] and AESN*GSYLQLVEISR and LAPLN*DSR from Fetuin [Swiss-Prot:P12763], were serially diluted to determine the linearity and accuracy of the self-build quantitative method. On the left are the results from manual calculation and on the right are the results from the self-build quantitative method. Glycopeptide concentration ratios calculated manually and those from the self-build method were all significantly associated with the theoretical values, with both correlation coefficients (*R*^*2*^) being >0.99. *denotes the N-glycosylation site.

The glycoproteins invertase and Fetuin were used as standards to evaluate the accuracy and feasibility of this integrated strategy for quantitation of N-glycoproteins. The glycopeptides were enriched from the glycoprotein standards with ConA lectin chromatography, and labeled with ^18^O and ^16^O. The ^18^O- and ^16^O-labeled glycopeptides were mixed in ratios of 1:1, 1:2, 2:1, 1:5, 5:1, 1:10, and 10:1, then analyzed by LC-MS. The relative concentration ratios of the glycopeptides (^18^O_3_/^16^O_3_) were calculated using Formula 1, and the relative concentration ratios of the non-glycopeptides (^18^O_2_/^16^O_2_) were calculated according to Formula 2 (see details in the methods section). In the mass spectrum of the mixed glycopeptides, the mass shift of 6 Da was easily identified for paired glycopeptides, and the mass shift of 4 Da was identified for paired non-glycopeptides (Additional file 2), accurately distinguishing between the two. Four glycopeptides and four non-glycopeptides in invertase and Fetuin were selected to further manually calculate relative concentration ratios. We found that peptide concentration ratios from manual calculation were similar to theoretical values (Table 1), and correlation coefficients (*R*^2^) were all >0.99 (Figure 2). These results indicated that our strategy had good linearity and accuracy in a 100-fold dynamic range.

**Table 1:** **Relative abundance ratios of the **^**18**^**O/**^**16**^**O-labeled glycopeptides and non-glycopeptides in glycoprotein standards**

**Protein**	**Peptide sequence**	**Expected ratio (Sample A:B)**	**Manually calculated ratio**	**Self-build quantitative method ratio**
Invertase	FATN*TTLTK	1 (1:1)	0.961	0.897
		2 (2:1)	1.997	1.982
		5 (5:1)	4.880	4.856
		0.5 (1:2)	0.563	0.491
		0.2 (1:5)	0.236	0.188
		0.1 (1:10)	0.129	0.104
		10 (10:1)	9.989	9.456
	LMTN*ETSDRPLVHFTPNK	1 (1:1)	0.992	0.953
		2 (2:1)	2.169	1.939
		5 (5:1)	4.980	5.033
		0.5 (1:2)	0.567	0.517
		0.2 (1:5)	0.216	0.192
		0.1 (1:10)	0.103	0.089
		10 (10:1)	10.110	10.050
	ENPYFTNR	1 (1:1)	0.937	0.997
		2 (2:1)	2.040	2.144
		5 (5:1)	5.217	5.462
		0.5 (1:2)	0.474	0.539
		0.2 (1:5)	0.177	0.208
		0.1 (1:10)	0.085	0.111
		10 (10:1)	10.558	11.160
	GLEDPEEYLR	1 (1:1)	0.769	0.837
		2 (2:1)	1.714	1.788
		5 (5:1)	5.014	4.437
		0.5 (1:2)	0.472	0.458
		0.2 (1:5)	0.184	0.183
		0.1 (1:10)	0.084	0.086
		10 (10:1)	9.636	11.110
Fetuin	AESN*GSYLQLVEISR	1 (1:1)	1.138	0.886
		2 (2:1)	1.735	1.588
		5 (5:1)	4.664	5.070
		0.5 (1:2)	0.562	0.424
		0.2 (1:5)	0.219	0.143
		0.1 (1:10)	0.075	0.036
		10 (10:1)	9.849	12.330
	LAPLN*DSR	1 (1:1)	0.980	0.965
		2 (2:1)	1.797	1.824
		5 (5:1)	4.627	4.174
		0.5 (1:2)	0.397	0.415
		0.2 (1:5)	0.158	0.184
		0.1 (1:10)	0.096	0.103
		10 (10:1)	11.289	N^*a*^
	ALGGEDVR	1 (1:1)	0.904	0.969
		2 (2:1)	2.116	1.913
		5 (5:1)	5.748	4.589
		0.5 (1:2)	0.469	0.471
		0.2 (1:5)	0.175	0.170
		0.1 (1:10)	0.088	0.080
		10 (10:1)	12.237	11.540
	TPIVGQPSIPGGPVR	1 (1:1)	1.067	1.042
		2 (2:1)	2.102	1.986
		5 (5:1)	5.823	5.559
		0.5 (1:2)	0.460	0.479
		0.2 (1:5)	0.183	0.163
		0.1 (1:10)	0.063	0.100
		10 (10:1)	10.414	10.120

*Denotes the N-glycosylation site.

^*a*^self-build quantitative method did not give a ratio.

Sample A was treated in ^18^O water and Sample B in ^16^O water. N = self-build quantitative method did not give a ratio.

### Confirmation of the precision of the self-build method

**Figure 3:**
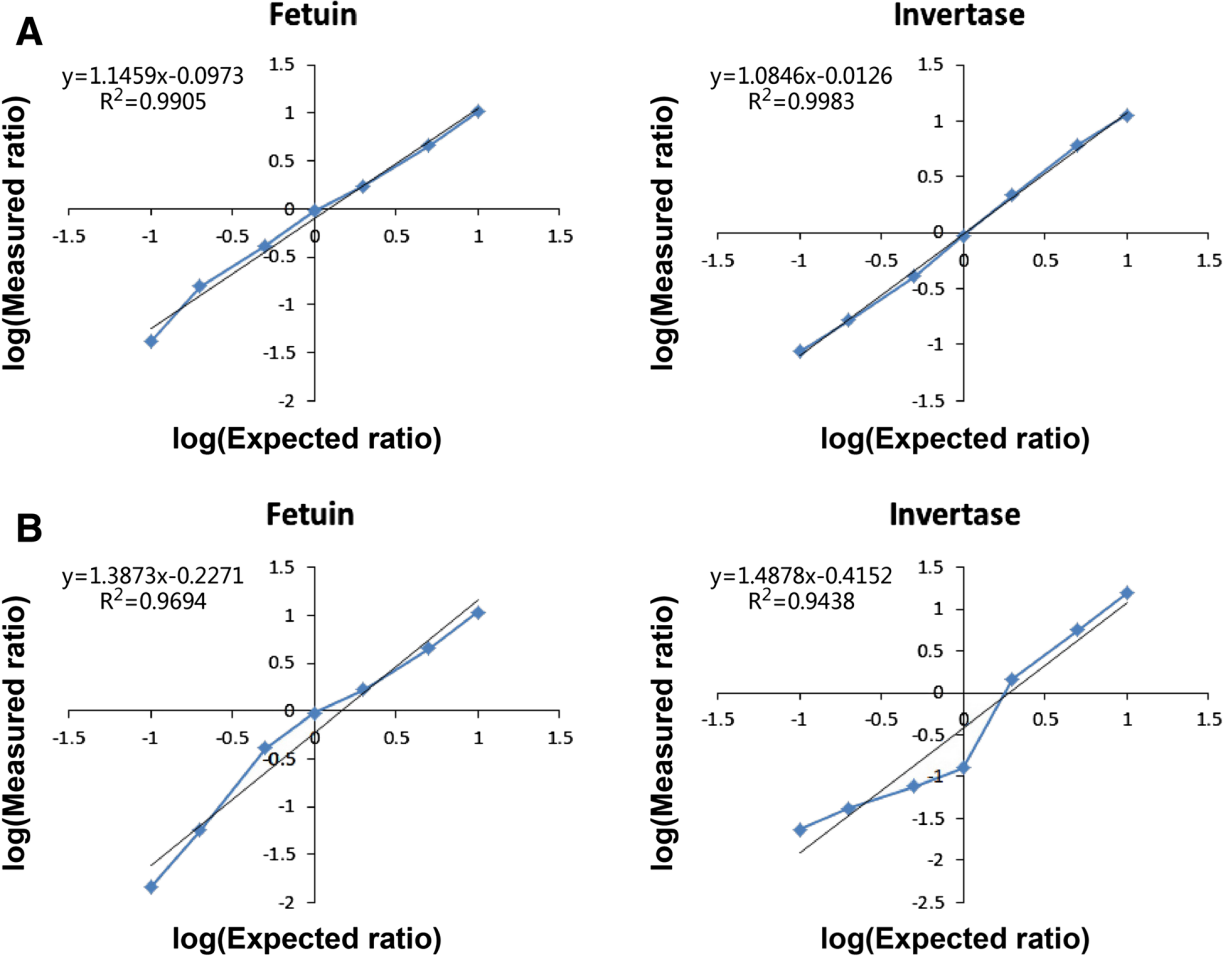
**Linearity and accuracy of the self-build quantitative method for glycoprotein standards.** The glycoprotein standards Fetuin and invertase were serially diluted to determine the linearity and accuracy of the self-build quantitative method. **(A)** The ratio of (Group A + Group B)/Group C. Glycopeptide concentration ratios calculated by the self-build method were significantly associated with the theoretical values, with both correlation coefficients (*R*^2^) being >0.99. **(B)** The ratio of Group A/Group C was calculated without considering back-labeling and incomplete labeling in glycoprotein standards; a difference was found between the result of the relative quantitation ratio of ^18^O/^16^O in **(B)** and the theoretical ratio.

Although data analysis of some ^18^O labeling methods can be supported by some automatic software, the tandem ^18^O_3_ labeling technique lacks matched software, and the data obtained has so far been analyzed with time-consuming manual calculation. Managing data from complex biological samples using manual calculation is difficult, necessitating an accurate, reliable, and user-friendly automated analysis method for data generated with ^18^O_3_ labeling [13]. In our study, two customized software packages, XPRESS and ASAPRatio, including the Trans-Proteomic Pipeline Ver. 4.5 (TPP, Seattle Proteome Center), were first used to quantitatively analyze data generated from the ^18^O_3_-labeled glycoprotein standards. The results were disappointing; XPRESS gave linear results to non-glycopeptides labeled with ^18^O_2_, rather than to the ^18^O_3_-labeled glycopeptides, and the quantitative results generated by ASAPRatio were even less satisfactory than those of XPRESS (Additional file 3). We established an automatic quantitative method for the ^18^O_3_ labeling technique based on Mascot Distiller and applied it to analyze the glycoprotein standard data obtained from LC-MS. As shown in Table 1, the peptide concentration ratios calculated by this quantitative method were similar to both the theoretical values and the manually calculated results, and had good linearity and accuracy within the ratio range of 1:10–10:1 (Table 1 and Figure 2). Similar results were found with the protein concentration ratios (Figure 3). These data indicate that this quantitative method is reliable for calculating concentration ratios of both peptides and proteins labeled with ^18^O_3_, and may replace the time-consuming manual calculation in time.

As mentioned in the methods section, three modification groups were defined in the self-build quantitative method, and the quantitative ratio of ^18^O/^16^O was finally generated via Formula 3 [(Group A + Group B)/Group C] to avoid the influences of back-exchange and incomplete C-terminal labeling on the final result. The ratio of Group A to Group C was used to evaluate the influences of back-exchange and incomplete C-terminal labeling. We found that if these influencing factors had not been excluded, the final quantitative ratios of ^18^O_3_/^16^O_3_ differed from the theoretical ones (Figure 3), demonstrating that even the labeling efficiency was improved in this study. These data indicate that the quantitative setting in our study is correct and can minimize the influence of incomplete labeling and back-exchange of ^18^O.

### Establishment of the quantitative criteria for this integrative strategy

In order to establish the measurement criteria for the relative quantification of glycoproteins and glycopeptides, the ^16^O/^18^O-labeled glycoprotein standard mixture at a ratio of 1:1 was repeatedly analyzed by LC-MS and quantitatively calculated seven times. The SD was detected in the spectrum of the glycoprotein standard, with a SD range of 0.023–0.186 for the glycopeptide and 0.075–0.216 for the glycoprotein. The relative quantitative ratios generated are listed in Table 2. An ^16^O/^18^O-labeled glycopeptide or glycoprotein ratio >3 times the SD value was considered a significant change; in contrast, when the ratio was within 1–3 times the SD, it was considered a minor change [13,43]. Thus, the quantitative criteria were defined as follows: Significant changes were determined when the ratio was smaller than 0.63 or greater than 1.57 for glycopeptides and less than 0.60 or over 1.65 for glycoproteins, whereas minor changes were assumed when the ratio was 0.63–0.84 or 1.19–1.57 for glycopeptides and 0.60–0.82 or 1.22–1.65 for glycoproteins.

**Table 2:** **The SD for glycoproteins and four glycopeptides from ConA-enriched Fetuin and invertase in a 1:1 **^**18**^**O/**^**16**^**O ratio calculated by the self-build quantitative method**

**Protein and peptide sequence**	**SD ratio**
**Invertase glycoprotein**	0.216
FATN*TTLTK	0.186
NPVLAAN*STQFR	0.156
**Fetuin glycoprotein**	0.075
LAPLN*DSR	0.054
AESN*GSYLQLVEISR	0.023

*denotes the N-glycosylation site.

### Validation of the feasibility of the integrative strategy in complex biological samples

Serum samples from three HCC patients and three healthy individuals were used to determine the feasibility of this strategy in complex clinical samples. Considering that gender and age may partially affect serum glycan distributions and a number of environmental variables (such as smoking) may also be associated with serum glycome components [44-46], we matched the HCC patients and healthy individuals as much as possible to decrease bias caused by individual differences.

The glycopeptides in the serum samples were separated and enriched with ConA, LCH, or WGA lectin chromatography, generating three subgroups of glycopeptides specifically recognized by ConA, LCH, and WGA, respectively. The glycopeptides were then labeled with ^18^O or ^16^O, followed by mixing the glycopeptides in a ratio of 1:1 from each subgroup of glycopeptides. Each mixture was repeatedly analyzed by LC-MS and quantitatively calculated seven times. We found that 44 unique glycopeptides and 30 glycoproteins with a specific glycan structure were differently expressed between HCC patients and healthy individuals (Additional file 4). Among these differentially expressed glycopeptides and glycoproteins, 14 and 13 changed in more than one lectin subgroup, respectively (see detailed data in Additional files 5 and 6). There were 67 unchanged glycopeptides in serum samples (see detailed data in Additional file 7). All N-linked glycopeptides had a consensus motif of Asn-X-Thr/Ser (X ≠ P). However, there were very low amounts of the differentially expressed glycopeptides/glycoproteins identified in our study, partially due to the limited volume of the serum samples and the multi-step processing of samples. All detailed data of detected glycopeptides in HCC patient and health control serum is shown in Additional file 8.

**Figure 4:**
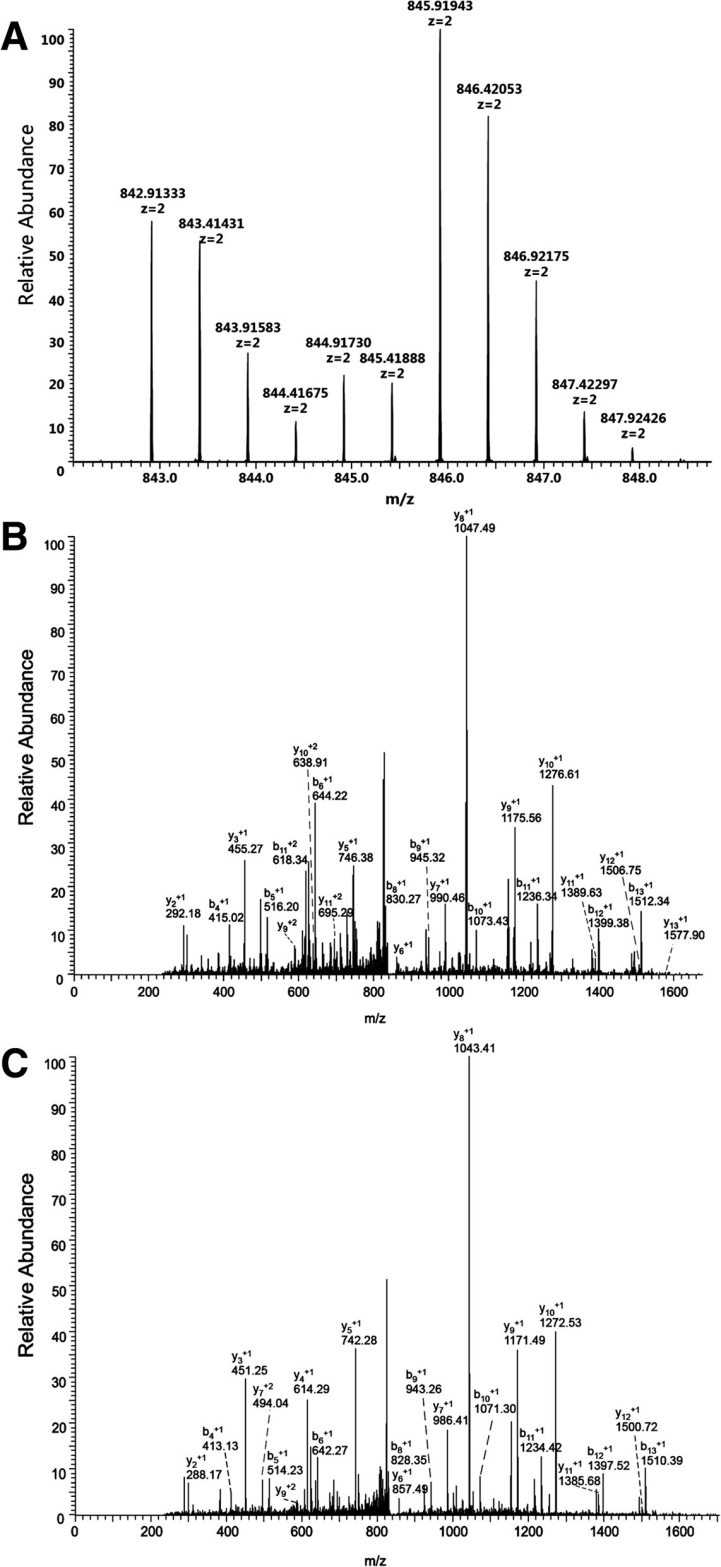
MS and quantitation of a deglycosylated peptide, LAN*LTQGEDQYYLR, from ConA-enriched CLUS [Swiss-Prot: P10909] glycoprotein in human serum. (A) Double-charged peptide labeled with ^18^O and ^16^O. The *m/z* 845.91943 (^18^O) and 842.91333 (^16^O) displays a 6 Da mass shift in the monoisotopic peaks; the intensity ratio of the ^18^O/^16^O isotope cluster was 1.812. **(B)** Mass spectrum of the glycopeptide labeled with ^18^O in *m/z* 845.91943. **(C)** Mass spectrum of the glycopeptide labeled with ^16^O in *m/z* 842.91333. *denotes the N-glycosylation site.

A representative Nano LC-ESI-MS/MS spectrum of a clusterin (CLUS) protein glycopeptide, LAN*LTQGEDQYYLR, in the ConA subgroup is shown in Figure 4; Figure 4A shows a magnified MS spectrum with a monoisotopic peak of double-charged peptide at *m/z* 845.91943 (^18^O) and 842.91333 (^16^O), representing a 6 Da mass shift. The MS spectrum indicated that there were three ^18^O atom labels and a mono-glycosylation site on this glycopeptide. The fragmented ion MS/MS spectrum had a mass shift of 117 Da between the y11 and y12 ions, equal to the mass shift generated by aspartic acid after being labeled by one ^18^O atom, and characteristically verified the deamidation of Asn in this position. The mass shift of 4 Da was displayed in all singly charged y ions (Figure 4B and C), indicating that the C-terminus was labeled by two ^18^O. A 2-Da mass shift was displayed in the b-ion series of monocharges, confirming that one ^18^O was present at the monoglycosylation site (Asn residue).

The quantitative results of the serum samples were verified again by manual calculation of the four selected glycopeptides. There was no significant difference between the automatically quantitated ratios and the manually calculated ones (Additional file 9), suggesting that this automatic quantitative method is reliable for analysis of complex biological samples.

**Figure 5:**
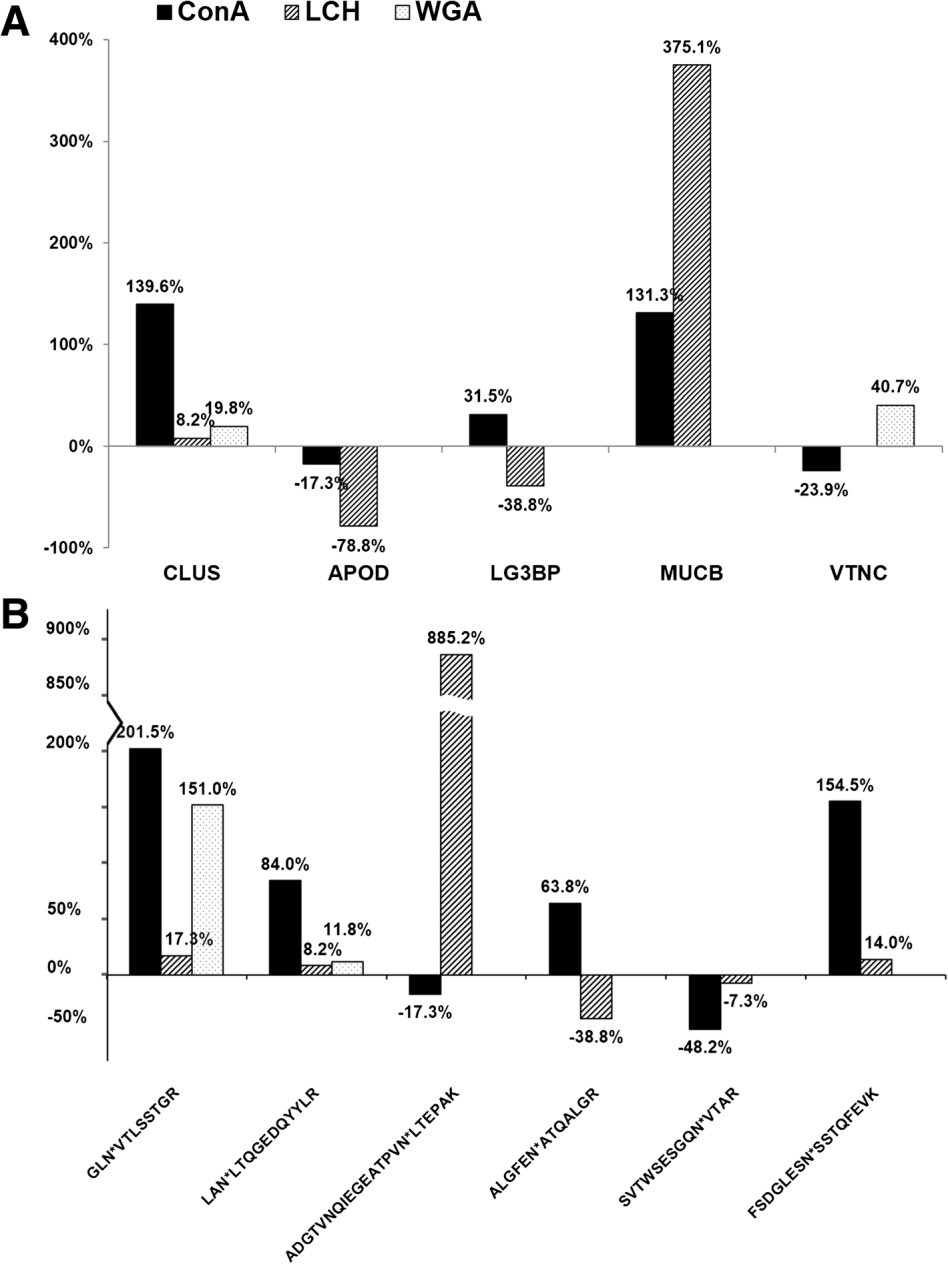
**Glycopeptides/glycoproteins with inconsistent changes in the ConA, LCH, and WGA subgroups. (A)** When comparing HCC patients with healthy individuals, five glycoproteins were altered inconsistently among the three lectin subgroups (ConA, LCH, and WGA); **(B)** When comparing the serum of HCC patients with that of healthy individuals, six glycopeptides changed discordantly among the three lectin subgroups (ConA, LCH, and WGA). Each column represents the percentage of the amount of glycopeptide/glycoprotein (HCC patients *versus* healthy individuals) in each lectin subgroup. Positive percentages denote upregulation, whereas negative percentages denote downregulation. Glycopeptide GLN*VTLSSTGR was from CO4A_HUMAN [Swiss-Prot:P0C0L4], LAN*LTQGEDQYYLR from CLUS_HUMAN [Swiss-Prot:P10909], ADGTVNQIEGEATPVN*LTEPAK from APOD_HUMAN [Swiss-Prot:P05090], ALGFEN*ATQALGR from LG3BP_HUMAN [Swiss-Prot:Q08380], SVTWSESGQN*VTAR from IGHA2_HUMAN [Swiss-Prot:P01877], and FSDGLESN*SSTQFEVK from CO4B_HUMAN [Swiss-Prot:P0C0L5]. Other glycoproteins were MUCB_HUMAN [Swiss-Prot:P04220] and VTNC_HUMAN [Swiss-Prot:P04004]. *denotes the N-glycosylation site.

**Figure 6:**
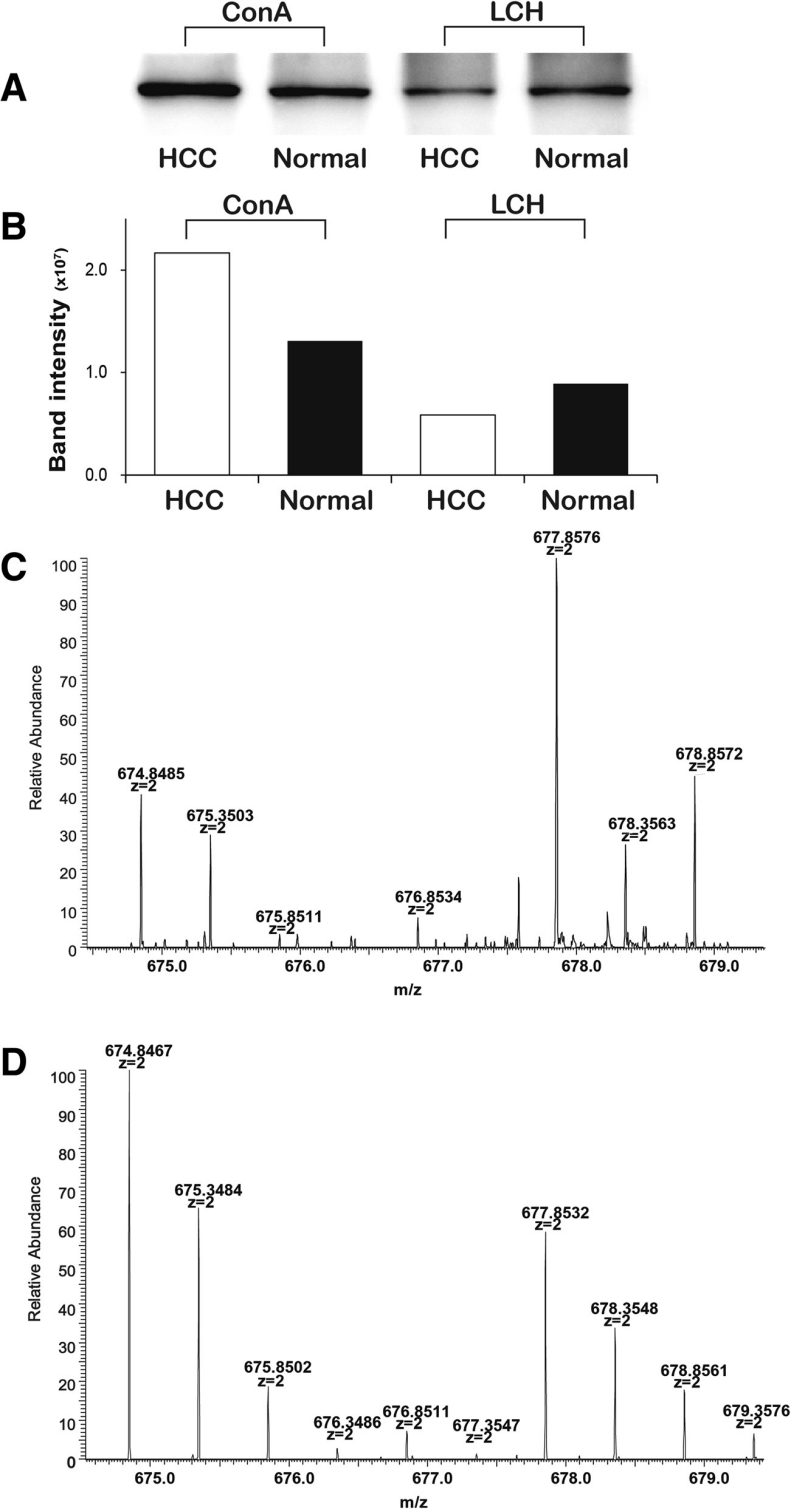
**Validation of glycoprotein LG3BP in different lectin subgroups by western blot. (A)** The expression levels of LG3BP in ConA and LCH lectin subgroups from the serum of HCC patients and healthy individuals were detected by western blot. **(B)** The target band intensities were quantified by ImageQuantTL software. The band intensity ratio of HCC patients *versus* healthy individuals was 1.66 in the ConA subgroup and 0.66 in the LCH subgroup. **(C)** The mass spectrum of a glycopeptide, ALGFEN*ATQALGR, from glycoprotein LG3BP in the ConA subgroups. **(D)** The mass spectrum of the glycopeptide, ALGFEN*ATQALGR, from glycoprotein LG3BP in the LCH subgroups. The ^18^O/^16^O ratios (HCC patients *versus* healthy individuals) of these glycopeptides were 1.64 in the ConA subgroup and 0.61 in the LCH subgroup. *denotes the N-glycosylation site.

Compared with the healthy individuals, the alterations of some glycopeptide/glycoprotein levels in HCC patients were inconsistent, even converse, among the three subgroups of glycopeptides (Figure 5). These data suggest that the glycan structure on specific glycosylation sites may also be altered in these glycoproteins. Compared with the studies using total serum or tissue glycoproteome, glycoprotein subgroups separated by lectin chromatography could reduce the complexity of tested samples and improve the detection of low-abundance proteins. Therefore, these glycan changes on specific glycoproteins may be sensitive potential biomarkers for disease diagnosis, which are worthy of further investigation. Among these proteins, apolipoprotein D (APOD) was down-regulated in HCC patient serum in all three lectin subgroups, and CLUS was up-regulated in all three lectin subgroups, consistent with previous data [47,48]. To further validate the quantitative results obtained by our strategy, we determined the expression levels of glycoprotein LG3BP by western blot in the ConA and LCH lectin subgroups from HCC patient and healthy individual serum. As shown in Figure 6, the band intensity ratio of HCC patients versus healthy individuals was 1.66 in the ConA subgroup and 0.66 in the LCH subgroup. These were very similar to the ratios of glycoproteins (1.32 in the ConA subgroup and 0.61 in the LCH subgroup) and glycopeptide ratios of the proteins (1.64 in the ConA subgroup and 0.61 in the LCH subgroup) obtained by our integrated strategy. The quantity changes observed in the integrated strategy were independently confirmed by western blot. These differentially expressed glycoproteins might play an important role in screening for sporadic HCC in the general population. All of the above results indicate that the present labeling strategy is feasible and reliable.

## Conclusions

In this study, we established an integrated research strategy for the high-throughput, quantitative analysis of N-linked glycoproteomics. This strategy integrated lectin chromatography and tandem ^18^O/^16^O labeling with LC-MS analysis and our novel automatic data analysis method. We also made modifications to the techniques used to avoid various interferences and enhance the labeling efficiency of ^18^O_3_. We demonstrated this strategy to be accurate and reliable using glycoprotein standards, and then identified a number of N-glycoproteins with specific glycan structures that were differently expressed between HCC patients and healthy individuals, as well as N-glycoproteins with modified glycosylation site occupancy. Western blot analysis further confirmed these results. This integrated strategy provides a useful tool for identifying disease-related N-glycosylation changes and glyco-biomarkers for diagnosis and prognosis of diseases.

## Methods

### Chemicals and materials

The ProteoMiner Protein Enrichment Kit was purchased from Bio-Rad (Hercules, CA), the PNGase-F from New England BioLabs (Ipswich, MA), the C18 cartridge from Waters (Milford, MA), the 3-kDa spin column from Millipore (Billerica, MA), and the immobilized trypsin beads from Applied Biosystems (Framingham, MA). The concanavalin A (ConA)-based and wheat germ agglutinin (WGA)-based glycoprotein isolation kit, the bicinchoninic acid (BCA) assay kit and MicroSpin column were obtained from Pierce (Rockford, IL). The LCH-based isolation kit was from GALAB (Germany). The glycoprotein standards (bovine Fetuin and yeast invertase), ^18^O water (97%), and other chemicals were obtained from Sigma-Aldrich (St. Louis, MO).

### Preparation of serum samples

The archived serum samples of patients with HCC were obtained from Zhongshan Hospital, Fudan University (Shanghai, China). Healthy individuals served as normal controls. Physiological conditions such as age, etc., were matched to decrease bias caused by individual differences. Detailed information regarding the HCC patients and controls were summarized in Additional file 10. This study was approved by the Research Ethics Committee of Zhongshan Hospital, and informed consent was obtained from all subjects.

The serum samples were stored at -80°C before processing. Equal volumes of serum from three HCC patients or three healthy individuals were pooled together to generate two sample pools, which were used in subsequent experiments. The most abundant serum proteins were removed by the ProteoMiner Protein Enrichment Kit according to the manufacturer's instruction. The protein concentrations were determined using the BCA assay kit.

### Digestion of glycoprotein standards and serum samples

The paired serum samples and the glycoprotein standards, Fetuin and yeast invertase, in solution were denatured at 100°C for 10 min. The samples were reduced with 10 mM dithiothreitol (DTT) at 57°C for 30 min and alkylated with 30 mM iodoacetamide at room temperature for 1 h in the dark. After desalting by spin column, the samples were digested with trypsin at an enzyme-to-substrate ratio of 1:50 (w/w) at 37°C for 16 h. To quench the trypsin and prevent back-exchange of ^18^O, the digested samples were boiled in a water bath for 10 min and then placed on ice for 5 min, as previously described [39].

### Lectin affinity chromatography

Lectin affinity chromatography was performed using ConA-, LCH-, and WGA-based isolation kits to separate out glycopeptides with specific glycan structure. Briefly, the digested serum samples or glycoprotein standards were diluted with binding/wash buffer and then added to the resin bed and incubated for 10 min at room temperature. The resin was then washed and the bound glycopeptides eluted and collected.

### Isotope labeling with ^18^O or ^16^O water

After lectin affinity chromatography, the peptides obtained from the samples and glycoprotein standards were desalted using SepPak C18 cartridges, and then dried in a vacuum centrifuge. The peptides were then mixed with immobilized trypsin (20% slurry v/w) for 20 min with gentle shaking, and then lyophilized. The lyophilized peptides were dissolved in 100 μL acetonitrile in 50 mM NH_4_HCO_3_ (pH 6.8) (ACN/NH_4_HCO_3_, 20% v/v) prepared with H_2_^16^O or H_2_^18^O in advance, then incubated at 37°C for 24 h to catalyze the labeling of tryptic peptides at the C-terminus. The immobilized trypsin beads were then removed by MicroSpin columns. A total of 5 μL formic acid was added to further inhibit any possible residual trypsin activity. The peptides were lyophilized and then dissolved in 100 mM NH_4_HCO_3_ buffer prepared in H_2_^16^O or H_2_^18^O. PNGase F was added at a concentration of 1 μL PNGase-F/mg of crude protein, and the labeling was conducted at 37°C overnight. Finally, the ^16^O- and ^18^O-labeled peptides were mixed at designated ratios (1:1, 2:1, 1:2, 5:1, 1:5, 10:1, and 1:10 for glycoprotein standards; 1:1 for samples) and lyophilized.

### Nano LC-electrospray ionization (ESI)-MS/MS

The lyophilized peptides were resuspended with 2% ACN in 0.1% formic acid, separated by nano LC, and then analyzed by online electrospray tandem mass spectrometry. The experiments were performed on a Nano Aquity UPLC system (Waters) connected to an LTQ Orbitrap XL mass spectrometer (Thermo Electron Corp., Bremen, Germany) interfaced with an online nano electrospray ion source (Michrom Bioresources, Auburn, CA). The peptide separation was performed in a Michrom CAPTRAP (500 μm i.d. × 2 mm trap column) and a Michrom C18 (3.5 μm, 100 μm i.d. × 15 cm reverse phase column) (Michrom Bioresources). The model glycoprotein digests (0.5 μg) were loaded onto the trap column and leached at a flow rate of 20 μL/min for 3 min. The mobile phases included 2% ACN in 0.1% formic acid (phase A and the loading phase) and 95% ACN in 0.1% formic acid (phase B). To achieve sufficient separation, a 60-min (for glycoprotein standards) or 90-min (for serum samples) linear gradient from 5% to 45% at phase B was employed. The flow rate of the mobile phase was set at 500 nL/min, and the electrospray voltage used was 1.6 kV. The linear gradient was adjusted to 90 min for serum samples analyses, while all other parameters remained unchanged. The LTQ Orbitrap XL mass spectrometer was operated in the data-dependent mode with an automatic switch between MS and MS/MS acquisition. The survey full-scan MS spectra with two microscans (m/z 350–1800) was acquired in Orbitrap at a resolution of 100,000 (at m/z 400) followed by eight MS/MS scans in LTQ trap. Dynamic exclusion was set to initiate a 60 s exclusion for ions analyzed twice within a 10 s interval.

### Manual calculation of relative concentration ratios

The mass spectra acquired by Nano LC-ESI-MS/MS of the samples were searched against the human International Protein Index (IPI) database (IPI human v3.45 FASTA with 71,983 entries, with bovine Fetuin and yeast invertase manually added), using the SEQUEST algorithm integrated into the Bioworks package (Version 3.3.1; Thermo Electron). The parameters for the SEQUEST search included: enzyme, partial trypsin; missed cleavages allowed, two; fixed modification, carboxyamidomethylation (Cys); variable modifications, deamidation (Asn +0.98 Da), deamidation plus ^18^O (Asn +2.98 Da), C-term (+4.01 Da), and oxidation (Met +15.99 Da); peptide tolerance, 10 ppm; and MS/MS tolerance, 1.00 Da. The statistical significance of the database search results was evaluated with the aid of PeptideProphet [49]. A minimum PeptideProphet probability score (P) filter of 0.9 was selected as a threshold to remove low-probability peptides.

The relative concentration ratios of the peptides were then manually calculated. Formula 1 was used to calculate the ratio (^16^O/^18^O) of glycopeptides as described previously [13]:

$$\text{ratio}\;\left(\frac{{}^{16}O}{{}^{18}O}\right)=\frac{I_{0}}{\begin{array}{l} I_{2}+I_{4}+I_{6}-\left(\frac{M_{2}}{M_{0}}\right)\;I_{4}-\left[\frac{M_{2}}{M_{0}}+\frac{M_{4}}{M_{0}}-{\left(\frac{M_{2}}{M_{0}}\right)}^{2}\right]\;I_{2}\\ \;-\;\left[\frac{M_{2}}{M_{0}}+\frac{M_{4}}{M_{0}}+\frac{M_{6}}{M_{0}}{\left(\frac{M_{2}}{M_{0}}\right)}^{2}+{\left(\frac{M_{2}}{M_{0}}\right)}^{3}-2\frac{M_{2}-M_{4}}{{M_{0}}^{2}}\right]\;I_{2} \end{array}}$$

Formula 2 was used to calculate the ratio (^16^O/^18^O) of the non-glycopeptides as described previously [50]:

$$\text{ratio}\;\left(\frac{{}^{16}O}{{}^{18}O}\right)=\frac{I_{0}}{I_{2}+I_{4}-\left(\frac{M_{2}}{M_{0}}\right)I_{2}-\left[\frac{M_{2}}{M_{0}}+\frac{M_{4}}{M_{0}}-{\left(\frac{M_{2}}{M_{0}}\right)}^{2}\right]\;I_{0}}$$

M_0_, M_2_, M_4_, and M_6_ are the corresponding theoretical relative intensities of the isotopic envelope of the peptide, calculated using MS-Isotope (http://prospector.ucsf.edu).

### Calculation of relative concentration ratios with the self-build quantitative method

The raw data acquired by Nano LC-ESI-MS/MS were searched against the Swiss-Prot database using the Mascot Distiller software (Version 2.3.2.0; Matrix Science) and user-defined search criteria. The search parameters were set according to the preceding settings of Bioworks. The relative concentration ratios were generated by the Mascot Distiller software with a self-build quantitative method. In this method, the quantitative protocol is the precursor. Taking into account the incomplete label or back-exchange on the C-terminus, three exclusive modification groups were used for calculating the ratios: Group A was comprised of two ^18^O labels on the C-terminus and one ^18^O label on each N-glycosylated Asn residue; Group B included one ^18^O label on the C-terminus and one ^18^O label on each N-glycosylated Asn residue; and Group C were labeled by ^16^O on both of these sites. As one given peptide may only carry one or another set of modifications, but never have a mixture of both sets, the “exclusive” modification group was used to avoid interference from the too-complex resultant data and too many variable modifications derived from the pooled samples. The isotope and impurity correction factors were set to 97% ^18^O based on actual use. The relative concentration ratios were calculated by Formula 3:

$$\text{Ratio}\;\left({}^{18}O{/}^{16}O\right)=\left(\text{Group}\;A+\text{Group}\;B\right)/\text{Group}\;C$$

The glycoprotein ratios were calculated according to the median of the glycopeptide ratios with the self-built quantitation software. Additional file 11 is self-build quantitation setting file and Additional file 12 is a modified unimod file.

### Western blot

The expression level of glycoprotein galectin-3-binding protein (LG3BP) was evaluated by western blot to validate the results of the integrated research strategy. The glycoproteins in the depleted pooled serum from three HCC patients and three healthy individuals were enriched by lectin affinity chromatography, and then separated by 10% sodium dodecyl sulfate polyacrylamide gel electrophoresis (SDS-PAGE) followed by transfer onto polyvinylidene fluoride membranes. Anti-LG3BP (Santa Cruz Biotechnology, Dallas, TX) was used as the primary antibody. The quantitative signals were acquired and quantified via a LAS-4000 imager and ImageQuantTL software (Version 7.0; GE Healthcare, Piscataway, NJ).

### Statistical evaluation

Based on the statistical three-sigma rule which states that nearly all values lie within 3 standard deviations (SD) of the mean for a normal distribution [51], we first established a set of criteria for statistical evaluation of glycopeptide/glycoprotein differences using the glycoprotein standards. Compared with the controls, a change of more than 3-fold of SD at an abundance ratio of 1.0 was considered statistically significant at a 99% confidence level [52,53].

## Abbreviations

AFP-L3: Lens culinaris affinitive alpha-fetoprotein; APOD: Apolipoprotein D; CLUS: Clusterin; ConA: Concanavalin A; DTT: Dithiothreitol; HCC: Hepatocellular carcinoma; LCH: Lens culinaris; LG3BP: Galectin-3-binding protein; WGA: Wheat germ agglutintin.

## Competing interests

The authors declare no conflict of interests with any company or financial organization.

## Authors’ contributions

JW carried out the experimental steps and wrote the paper; JW and CZ were involved in serum peptide purification; JW, WZ, JY and HJL performed the mass spectrometric analysis of proteins; CZ and QZD were involved in serum samples and clinical data collection; HJZ and LXQ designed the experiments and supervised the research manuscript. All authors read and approved the manuscript.

## Supplementary Material

Additional file 1 **The reaction of three **^**18**^**O atoms labeling happened in the glycopeptides by catalysis with Trypsin and PNGase F.** The reaction is reversible at the ^18^O labeling of the C-terminal catalyzed with trypsin, indicating that the back-exchange and C-terminal single ^18^O labeling in the C-terminal cannot be completely avoided in the reaction product. This feature is a problem identified in the experimental operation, result analysis, and quantitative method design, but do not need to consider in the labeling process of PNGase-F catalysis. Click here for file

Additional file 2 **The mass shifts identified by mass spectrum between paired **^**18**^**O and **^**16**^**O labeled glycopeptides/non-glycopeptides from standard glycoprotein Invertase.** (A) For the non-glycopeptides VFWYEPSQK, the mass shift of 4 Da was generated in mass spectrometry. (B) For the glycopeptide FATN*TTLTK, the mass shift of 6 Da was generated in mass spectrometry. *denotes the N-glycosylation site. Click here for file

Additional file 3 ASAP and XPRESS ratios of glycopeptides and non-glycopeptides in the dynamic range of 1:10–10:1 derived by Trans-Proteomic Pipeline Ver. 4.5. Click here for file

Additional file 4 **The number of differently expressed glycoproteins/glycopeptides between HCC patients and healthy individuals in the three lectin subgroups.** The data calculated by self-build quantitative method. Click here for file

Additional file 5 **The table of changed glycopeptides in HCC patient serum (**^**18**^**O Labeling) compared to health control (**^**16**^**O Labeling).** Click here for file

Additional file 6 **The table of changed glycoproteins in HCC patient serum (**^**18**^**O Labeling) compared to health control (**^**16**^**O Labeling).** Click here for file

Additional file 7 **The table of unchanged glycopeptides in HCC patient serum (**^**18**^**O Labeling) compared to health control (**^**16**^**O Labeling).** Click here for file

Additional file 8 **The detailed data of detected glycopeptides in HCC patient serum (**^**18**^**O Labeling) compared to health control (**^**16**^**O Labeling).** Click here for file

Additional file 9 **Calculation of abundance ratios of four glycopeptides between HCC patients and healthy individuals.** The ratios calculated manually were similar to the ratios obtained by self-build quantitative method, which indicated the reliability of the quantitative results of our integrated research strategy. Click here for file

Additional file 10 Physiological/pathological characteristics of patients and healthy individuals enrolled in this study. Click here for file

Additional file 11 Our self-build quantitation setting file. Click here for file

Additional file 12 **A Modified unimod file.** You can download Additional files 9 and 10, copy them into the directory of /mascot/config/, please backup the original files before copying. Click here for file
