# Supplementary material for: An integrative strategy for quantitative analysis of the N-glycoproteome in complex biological samples
Source: Proteome Sci. 2014 Jan 15;12:4. doi: 10.1186/1477-5956-12-4 (PMC3923275; doi:10.1186/1477-5956-12-4)

Additional file 1: The reaction of three ^{18}O atoms labeling happened in the glycopeptides by catalysis with Trypsin and PNGase F. The reaction is reversible at the ^{18}O labeling of the C-terminal catalyzed with trypsin, indicating that the back-exchange and C-terminal single ^{18}O labeling in the C-terminal cannot be completely avoided in the reaction product. This feature is a problem identified in the experimental operation, result analysis, and quantitative method design, but do not need to consider in the labeling process of PNGase-F catalysis.

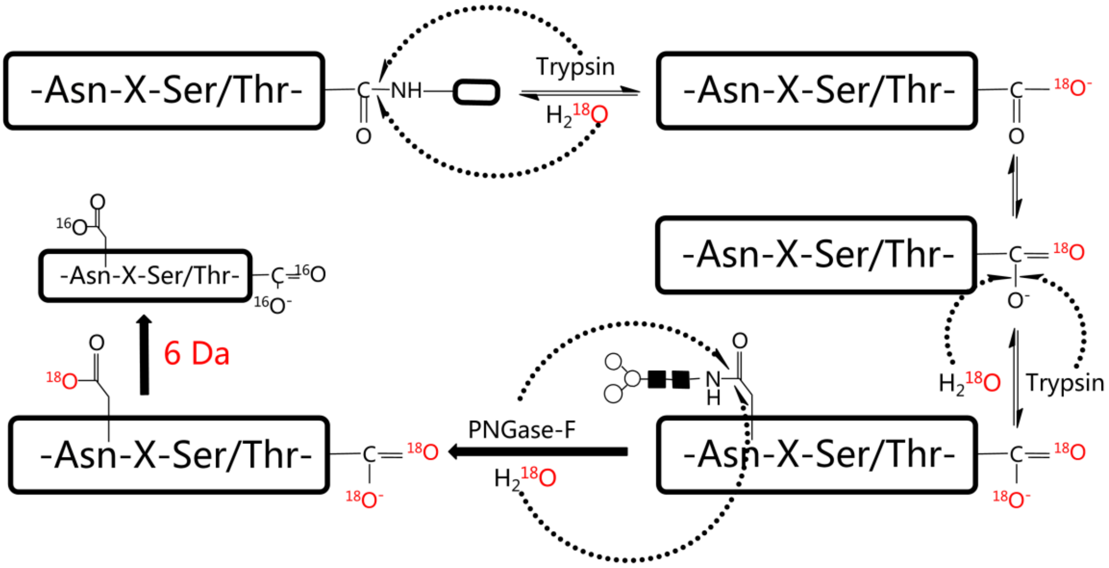

Supplement: Additional file 1 — The reaction of three 18O atoms labeling happened in the glycopeptides by catalysis with Trypsin and PNGase F. The reaction is reversible at the 18O labeling of the C-terminal catalyzed with trypsin, indicating that the back-exchange and C-terminal single 18O labeling in the C-terminal cannot be completely avoided in the reaction product. This feature is a problem identified in the experimental operation, result analysis, and quantitative method design, but do not need to consider in the labeling process of PNGase-F catalysis. [file 1477-5956-12-4-S1.pdf]
