# Supplementary material for: An integrative strategy for quantitative analysis of the N-glycoproteome in complex biological samples
Source: Proteome Sci. 2014 Jan 15;12:4. doi: 10.1186/1477-5956-12-4 (PMC3923275; doi:10.1186/1477-5956-12-4)

Additional file 2: The mass shifts identified by mass spectrum between paired ^{18}O and ^{16}O labeled glycopeptides/non-glycopeptides from standard glycoprotein Invertase. (A) For the non-glycopeptides VFWYEPSQK, the mass shift of 4 Da was generated in mass spectrometry. (B) For the glycopeptide FATN*TTLTK, the mass shift of 6 Da was generated in mass spectrometry. * denotes the N-glycosylation site.

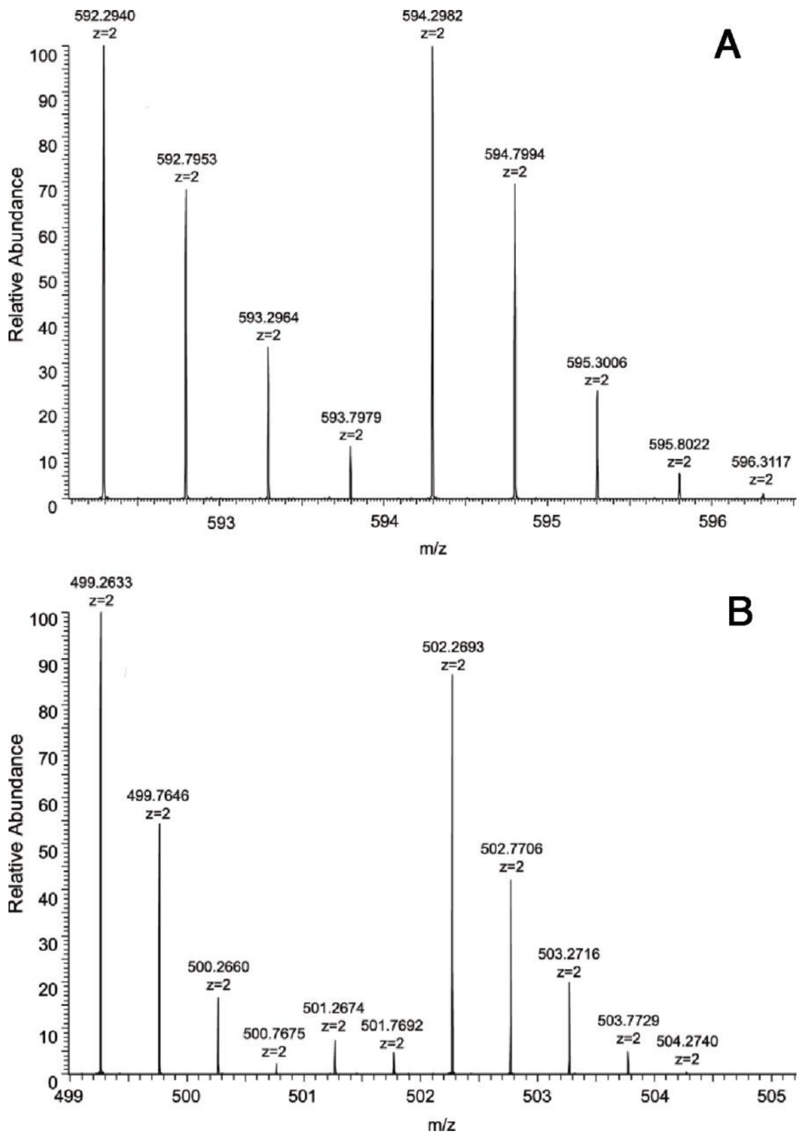

Supplement: Additional file 2 — The mass shifts identified by mass spectrum between paired 18 O and 16 O labeled glycopeptides/non-glycopeptides from standard glycoprotein Invertase. (A) For the non-glycopeptides VFWYEPSQK, the mass shift of 4 Da was generated in mass spectrometry. (B) For the glycopeptide FATN*TTLTK, the mass shift of 6 Da was generated in mass spectrometry. *denotes the N-glycosylation site. [file 1477-5956-12-4-S2.pdf]
