# Supplementary material for: An integrative strategy for quantitative analysis of the N-glycoproteome in complex biological samples
Source: Proteome Sci. 2014 Jan 15;12:4. doi: 10.1186/1477-5956-12-4 (PMC3923275; doi:10.1186/1477-5956-12-4)

Additional file 3: ASAP and XPRESS ratios of glycopeptides and non-glycopeptides in the dynamic range of 1:10–10:1 derived by Trans-Proteomic Pipeline Ver. 4.5 (TPP, Seattle Proteome Center). * denotes the N-glycosylation site. Glycopeptide FATN*TTLTK from Invertase [Swiss-Prot: P00724], LAPLNDSR from Fetuin [Swiss-Prot: P12763], peptide GLEDPEEYLR from Invertase [Swiss-Prot: P00724], ALGGEDVR from Fetuin [Swiss-Prot: P12763].

(A) XPRESS

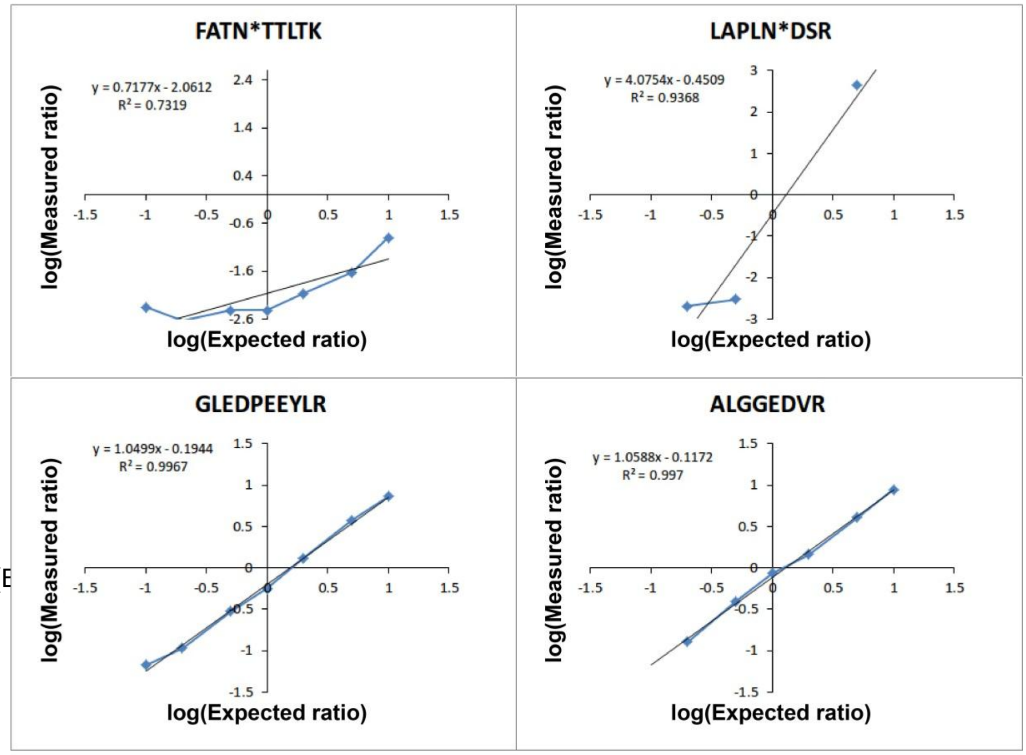

(B) ASAP ratio

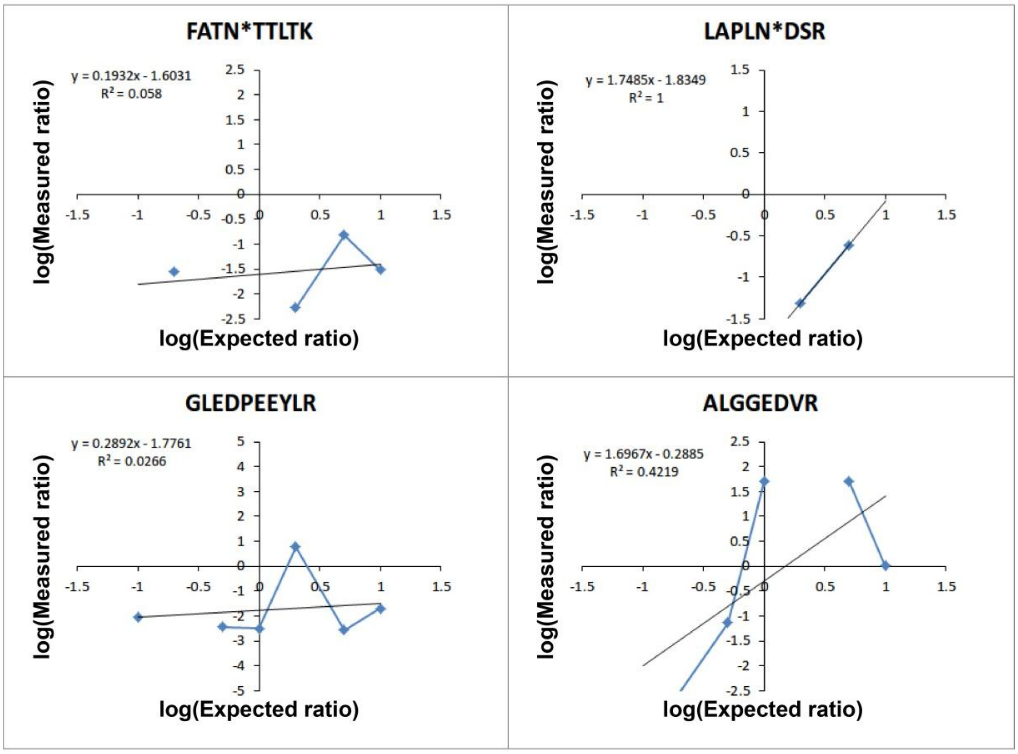

Supplement: Additional file 3 — ASAP and XPRESS ratios of glycopeptides and non-glycopeptides in the dynamic range of 1:10–10:1 derived by Trans-Proteomic Pipeline Ver. 4.5. [file 1477-5956-12-4-S3.pdf]
