# Supplementary material for: An integrative strategy for quantitative analysis of the N-glycoproteome in complex biological samples
Source: Proteome Sci. 2014 Jan 15;12:4. doi: 10.1186/1477-5956-12-4 (PMC3923275; doi:10.1186/1477-5956-12-4)
Supplement: Additional file 4 — The number of differently expressed glycoproteins/glycopeptides between HCC patients and healthy individuals in the three lectin subgroups. The data calculated by self-build quantitative method. [file 1477-5956-12-4-S4.pdf]

Additional file 4: The number of differently expressed glycoproteins/glycopeptides between HCC patients and healthy individuals in the three lectin subgroups. The data calculated by self-build quantitative method.

Lectin subgroup	Changed glycoprotein	Significantly changed glycoprotein	Changed glycopeptide	Significantly changed glycopeptide
ConA	17	14	26	21
LCH	8	5	12	7
WGA	5	4	6	4
