# Supplementary material for: An integrative strategy for quantitative analysis of the N-glycoproteome in complex biological samples
Source: Proteome Sci. 2014 Jan 15;12:4. doi: 10.1186/1477-5956-12-4 (PMC3923275; doi:10.1186/1477-5956-12-4)
Supplement: Additional file 5 — The table of changed glycopeptides in HCC patient serum (18O Labeling) compared to health control (16O Labeling). [file 1477-5956-12-4-S5.pdf]

Additional file 5: The table of changed glycopeptides in HCC patient serum (^{18}O Labeling) compared to health control (^{16}O Labeling). * denotes the N-glycosylation site.

Lectin subgroup	Swiss-Prot	Protein Entry name	Peptide Sequence	N-sites	Peptide Position	$^{18}O/^{16}O$ Ratio	SD
ConA	P04114	APOB_HUMAN	FVEGSHN*STVSLTTK	3411	3405~3419	0.093	0.01
ConA	P01860	IGHG3_HUMA	EEQYN*STFR	227	223~231	0.281	0.05
ConA	Q6S9Z5	ZN474_HUMA	HSN*SSDHLTGLK	200	198~209	0.438	0.16
ConA	P01877	IGHA2_HUMA	SVTWSESGQN*VTAR	47	38~51	0.518	0.04
ConA	P04114	APOB_HUMAN	SN*ASSTDSASYYPLTGDTR	983	982~1000	0.714	0.13
ConA	P04004	VTNC_HUMAN	NN*ATVHEQVGGPSLTSDLQAQSK	86	85~107	0.761	0.11
ConA	P01857	IGHG1_HUMA	EEQYN*STYR	180	176~184	0.809	0.03
ConA	P05090	APOD_HUMAN	ADGTVNQIEGEATPVN*LTEPAK	98	83~104	0.828	0.07
ConA	P04114	APOB_HUMAN	FN*SSYLQGTNQITGR	1523	1522~1536	1.384	0.36
ConA	Q08380	LG3BP_HUMA	ALGFEN*ATQALGR	69	64~76	1.638	0.55
ConA	P04114	APOB_HUMAN	FEVDSPVYN*ATWSASLK	3895	3887~3903	1.695	0.07
ConA	P02751	FINC_HUMAN	LDAPTNLQFVN*ETDSTVLVR	1007	997~1016	1.802	0.08
ConA	P10909	CLUS_HUMAN	LAN*LTQGEDQYYLR	374	372~385	1.840	0.04
ConA	P04278	SHBG_HUMAN	LDVDQALN*R	380	373~381	2.203	0.28
ConA	P02675	FIBB_HUMAN	ALMDGASQLMGEN*R	394	382~395	2.366	0.03
ConA	P0C0L5	CO4B_HUMAN	FSDGLESN*SSTQFEVK	226	219~234	2.545	0.09
ConA	P02675	FIBB_HUMAN	LMDGASQLMGEN*R	394	383~395	2.771	0.17
ConA	P07357	CO8A_HUMAN	GGSSGWSGGLAQN*R	437	425~438	2.800	0.63
ConA	P0C0L4	CO4A_HUMAN	GLN*VTLSSTGR	1328	1326~1336	3.015	0.07
ConA	P04220	MUCB_HUMA	THTN*ISESHPN*ATFS	210, 217	207~221	3.087	0.11
ConA	P02675	FIBB_HUMAN	DGASQLMGEN*R	394	385~395	3.270	0.29
ConA	P02675	FIBB_HUMAN	GTAGNALMDGASQLMGEN*R	394	377~395	3.615	0.50
ConA	P02675	FIBB_HUMAN	MDGASQLMGEN*R	394	384~395	4.044	0.20
ConA	P10909	CLUS_HUMAN	QLEEFLN*QSSPF	145	139~150	6.057	0.25
ConA	P07996	TSP1_HUMAN	VVN*STTGPGEHLR	1067	1065~1077	7.344	0.10
ConA	P55056	APOC4_HUMA	ELLETVVN*R	63	56~64	11.410	0.57
LCH	P55058	PLTP_HUMAN	MLQITN*ASLGLR	94	89~100	0.168	0.32
LCH	P05090	APOD_HUMAN	QIEGEATPVN*LTEPAK	98	89~104	0.212	0.08
LCH	Q08380	LG3BP_HUMA	ALGFEN*ATQALGR	69	64~76	0.612	0.03
LCH	P01857	IGHG1_HUMA	EEQYN*STYR	180	176~184	0.639	0.03
LCH	P00450	CERU_HUMAN	EHEGAIYPDN*TTDFQR	138	129~144	0.816	0.10
LCH	P33151	CADH5_HUMA	EVYPWYN*LTVEAK	442	436~448	0.834	0.16
LCH	P08603	CFAH_HUMAN	SQPPQIEHGTIN*SSR	882	871~885	0.836	0.08
LCH	O75636	FCN3_HUMAN	VELEDFNGN*R	189	171~190	1.304	0.04
LCH	P04220	MUCB_HUMA	THTNISESHPN*ATFS	217	207~221	1.702	0.06
LCH	P04220	MUCB_HUMA	THTNISESHPN*ATFSAVG	217	207~224	4.998	0.13
LCH	P04220	MUCB_HUMA	GLTFQQN*ASSMCGPDQDTAIRV	147	141~162	8.834	0.06
LCH	P05090	APOD_HUMAN	ADGTVNQIEGEATPVN*LTEPAK	98	83~104	9.852	0.28
WGA	Q96QU1	PCD15_HUMA	QMLFLN*STGR	97	92~101	0.480	0.04
WGA	Q9H9A7	RMI1_HUMAN	FSHNPN*TTNN	357	352~361	39.27	0.04
WGA	Q01081	U2AF1_HUMA	KVN*CSFYFK	17	15~23	0.500	0.02
WGA	P10909	CLUS_HUMAN	LAN*LTQGEDQYYLR	374	372~385	1.198	0.06
WGA	P04004	VTNC_HUMAN	NN*ATVHEQVGGPSLTSDLQAQSK	86	85~107	1.407	0.02
WGA	P0C0L4	CO4A_HUMAN	GLN*VTLSSTGR	1328	1326~1336	2.510	0.26
