# Supplementary material for: An integrative strategy for quantitative analysis of the N-glycoproteome in complex biological samples
Source: Proteome Sci. 2014 Jan 15;12:4. doi: 10.1186/1477-5956-12-4 (PMC3923275; doi:10.1186/1477-5956-12-4)
Supplement: Additional file 6 — The table of changed glycoproteins in HCC patient serum (18O Labeling) compared to health control (16O Labeling). [file 1477-5956-12-4-S6.pdf]

Additional file 6: The table of changed glycoproteins in HCC patient serum (^{18}O Labeling) compared to health control (^{16}O Labeling).

Lectin subgroup	Accession ID （Swiss-Prot）	Entry name	Protein description	$^{18}O/^{16}O$ Ratio
**ConA**	P01860	IGHG3_HUMAN	Ig gamma-3 chain C region OS=Homo sapiens GN=IGHG3 PE=1 SV=2	0.25
**ConA**	P04114	APOB_HUMAN	Apolipoprotein B-100 OS=Homo sapiens GN=APOB PE=1 SV=2	0.31
**ConA**	Q6S9Z5	ZN474_HUMAN	Zinc finger protein 474 OS=Homo sapiens GN=ZNF474 PE=2 SV=1	0.44
**ConA**	P01877	IGHA2_HUMAN	Ig alpha-2 chain C region OS=Homo sapiens GN=IGHA2 PE=1 SV=3	0.53
**ConA**	P04004	VTNC_HUMAN	Vitronectin OS=Homo sapiens GN=VTN PE=1 SV=1	0.76
**ConA**	Q08380	LG3BP_HUMAN	Galectin-3-binding protein OS=Homo sapiens GN=LGALS3BP PE=1 SV=1	1.32
**ConA**	P02751	FINC_HUMAN	Fibronectin OS=Homo sapiens GN=FN1 PE=1 SV=4	1.51
**ConA**	O75636	FCN3_HUMAN	Ficolin-3 OS=Homo sapiens GN=FCN3 PE=1 SV=2	2.09
**ConA**	P04278	SHBG_HUMAN	Sex hormone-binding globulin OS=Homo sapiens GN=SHBG PE=1 SV=2	2.20
**ConA**	P04220	MUCB_HUMAN	Ig mu heavy chain disease protein OS=Homo sapiens PE=1 SV=1	2.31
**ConA**	P10909	CLUS_HUMAN	Clusterin OS=Homo sapiens GN=CLU PE=1 SV=1	2.40
**ConA**	P07357	CO8A_HUMAN	Complement component C8 alpha chain OS=Homo sapiens GN=C8A PE=1 SV=2	2.44
**ConA**	P0C0L5	CO4B_HUMAN	Complement C4-B OS=Homo sapiens GN=C4B PE=1 SV=1	2.96
**ConA**	P0C0L4	CO4A_HUMAN	Complement C4-A OS=Homo sapiens GN=C4A PE=1 SV=2	3.07
**ConA**	P02675	FIBB_HUMAN	Fibrinogen beta chain OS=Homo sapiens GN=FGB PE=1 SV=2	3.24
**ConA**	P07996	TSP1_HUMAN	Thrombospondin-1 OS=Homo sapiens GN=THBS1 PE=1 SV=2	7.34
**ConA**	P55056	APOC4_HUMAN	Apolipoprotein C-IV OS=Homo sapiens GN=APOC4 PE=1 SV=1	11.41
**LCH**	P55058	PLTP_HUMAN	Phospholipid transfer protein OS=Homo sapiens GN=PLTP PE=1 SV=1	0.17
**LCH**	P05090	APOD_HUMAN	Apolipoprotein D OS=Homo sapiens GN=APOD PE=1 SV=1	0.21
**LCH**	Q08380	LG3BP_HUMAN	Galectin-3-binding protein OS=Homo sapiens GN=LGALS3BP PE=1 SV=1	0.61
**LCH**	P08603	CFAH_HUMAN	Complement factor H OS=Homo sapiens GN=CFH PE=1 SV=4	0.66
**LCH**	P01857	IGHG1_HUMAN	Ig gamma-1 chain C region OS=Homo sapiens GN=IGHG1 PE=1 SV=1	0.67
**LCH**	O75636	FCN3_HUMAN	Ficolin-3 OS=Homo sapiens GN=FCN3 PE=1 SV=2	2.09
**LCH**	P01871	IGHM_HUMAN	Ig mu chain C region OS=Homo sapiens GN=IGHM PE=1 SV=3	4.51
**LCH**	P04220	MUCB_HUMAN	Ig mu heavy chain disease protein OS=Homo sapiens PE=1 SV=1	4.75
**WGA**	Q96QU1	PCD15_HUMAN	Protocadherin-15 OS=Homo sapiens GN=PCDH15 PE=1 SV=2	0.48
**WGA**	Q01081	U2AF1_HUMAN	Splicing factor U2AF 35 kDa subunit OS=Homo sapiens GN=U2AF1 PE=1 SV=3	0.50
**WGA**	P04004	VTNC_HUMAN	Vitronectin OS=Homo sapiens GN=VTN PE=1 SV=1	1.41
**WGA**	P0C0L4	CO4A_HUMAN	Complement C4-A OS=Homo sapiens GN=C4A PE=1 SV=2	2.51
**WGA**	Q9H9A7	RMI1_HUMAN	RecQ-mediated genome instability protein 1 OS=Homo sapiens GN=RMI1 PE=1 SV=3	39.27
