# Supplementary material for: An integrative strategy for quantitative analysis of the N-glycoproteome in complex biological samples
Source: Proteome Sci. 2014 Jan 15;12:4. doi: 10.1186/1477-5956-12-4 (PMC3923275; doi:10.1186/1477-5956-12-4)
Supplement: Additional file 7 — The table of unchanged glycopeptides in HCC patient serum (18O Labeling) compared to health control (16O Labeling). [file 1477-5956-12-4-S7.pdf]

Additional file 7: The table of unchanged glycopeptides in HCC patient serum (^{18}O Labeling) compared to health control (^{16}O Labeling). * denotes the N-glycosylation site.

Lectin subgroup	Swiss-Prot	Protein Entry name	Peptide Sequence	N-sites	Peptide Position	$^{18}O/^{16}O$ Ratio	SD
ConA	P02745	C1QA_HUMAN	DTVITNQEEPYQN*HSGR	146	134~150	1.056	0.10
ConA	P08603	CFAH_HUMAN	SQPPQIEHGTIN*SSR	882	871~885	1.163	0.08
ConA	P0C0L5	CO4B_HUMAN	DGLESN*SSTQFEVK	226	217~234	1.059	0.23
ConA	P02675	FIBG_HUMAN	DLQSLEDILHQVEN*K	78	65~79	1.038	0.21
ConA	P02675	FIBG_HUMAN	VDKDLQSLEDILHQVEN*K	78	62~79	0.904	0.09
ConA	P02751	FINC_HUMAN	VN*ETDSTVLVR	1007	1006~1016	1.068	0.09
ConA	P01859	IGHG2_HUMAN	EEQFN*STFR	176	172~180	1.1605	0.13
ConA	P01871	IGHM_HUMAN	THTNISESHPN*ATF	217	207~220	0.924	0.34
ConA	P01871	IGHM_HUMAN	THTNISESHPN*ATFSAVG	217	207~224	1.16	0.07
ConA	P01591	IGJ_HUMAN	EN*ISDPTSPLR	71	70~80	1.107	0.30
ConA	P48740	MASP1_HUMAN	FGYILHTDN*R	178	170~179	0.8522	0.19
ConA	Q96QU1	PCD15_HUMAN	QMLFLN*STGR	97	92~101	0.90965	0.02
ConA	P00734	THRB_HUMAN	GHVN*ITR	121	118~124	1.1665	0.02
LCH	Q8NI99	ANGL6_HUMAN	VLN*ASAEAQR	145	143~152	0.9258	0.02
LCH	P04114	APOB_HUMAN	FEVDSPVYN*ATWSASLK	3895	3887~3903	0.95805	0.04
LCH	P04114	APOB_HUMAN	FN*SSYLQGTNQITGR	1523	1522~1536	0.9329	0.04
LCH	P04114	APOB_HUMAN	FVEGSHN*STVSLTTK	3411	3405~3419	1.0287	2.50
LCH	P04114	APOB_HUMAN	SN*ASSTDSASYYPLTGDTR	983	982~1000	0.9878	0.18
LCH	P04114	APOB_HUMAN	SNLYN*WSASYSGGNTSTDHFSLR	1368	1364~1386	1.13915	0.01
LCH	P05090	APOD_HUMAN	ATPVN*LTEPAK	98	94~104	1.167	0.35
LCH	P02745	C1QA_HUMAN	DTVITNQEEPYQN*HSGR	146	134~150	1.118	0.20
LCH	P02745	C1QA_HUMAN	QEEPYQN*HSGR	146	140~150	0.845	0.06
LCH	P02745	C1QA_HUMAN	TVITNQEEPYQN*HSGR	146	135~150	1.125	0.40
LCH	P02745	C1QA_HUMAN	VVIFDTVITNQEEPYQN*HSGR	146	130~150	1.082	0.49
LCH	P00450	CERU_HUMAN	EGAIYPDN*TTDFQR	138	131~144	0.852	0.20
LCH	P10909	CLUS_HUMAN	EDALN*ETR	86	82~89	1.1017	0.18
LCH	P10909	CLUS_HUMAN	LAN*LTQGEDQYYLR	374	372~385	0.8474	0.08
LCH	P0C0L4	CO4A_HUMAN	GLN*VTLSSTGR	1328	1326~1336	1.0893	0.26
LCH	P0C0L5	CO4B_HUMAN	DGLESN*SSTQFEVK	226	217~234	0.885	0.29
LCH	P0C0L5	CO4B_HUMAN	FSDGLESN*SSTQFEVK	226	219~234	0.9425	0.32
LCH	P02748	CO9_HUMAN	AVN*ITSENLIDDVVSLIR	415	413~430	0.9298	0.04
LCH	P23142	FBLN1_HUMAN	GDN*ASLEATFVK	98	96~107	1.1255	0.31
LCH	P02675	FIBB_HUMAN	ALMDGASQLMGEN*R	394	382~395	1.101	0.34
LCH	P02675	FIBB_HUMAN	GTAGNALMDGASQLMGEN*R	394	377~395	1.1171	0.19
LCH	P02675	FIBG_HUMAN	DLQSLEDILHQVEN*K	78	65~79	0.8615	0.11
LCH	P02675	FIBG_HUMAN	SLEDILHQVEN*K	78	68~79	1.027	0.03
LCH	P02675	FIBG_HUMAN	VDKDLQSLEDILHQVEN*K	78	62~79	0.86776	0.10
LCH	O95714	HERC2_HUMAN	SLTGASGN*ASSLPGVEALVGWLLD	2488	2481~2504	0.8662	0.10
LCH	P01877	IGHA2_HUMAN	SVTWSESGQN*VTAR	47	38~51	1.134	0.39
LCH	P01877	IGHA2_HUMAN	TPLTAN*ITK	205	200~208	0.9907	0.38

LCH	P01859	IGHG2_HUMAN	EEQFN*STFR	176	172~180	0.988	0.44
LCH	P01860	IGHG3_HUMAN	EEQYN*STFR	227	223~231	0.97985	0.26
LCH	P01871	IGHM_HUMAN	GLTFQQN*ASSM	147	141~151	1.1268	0.26
LCH	P01871	IGHM_HUMAN	THTNISESHPN*AT	217	207~219	1.1655	0.15
LCH	P01871	IGHM_HUMAN	THTNISESHPN*ATFSAVGE	217	207~225	0.9991	0.12
LCH	P01591	IGJ_HUMAN	EN*ISDPTSPLR	71	70~80	0.94345	0.02
LCH	Q08380	LG3BP_HUMAN	AAIPSALDTN*SSK	551	542~554	0.85375	0.01
LCH	Q07954	LRP1_HUMAN	IETILLN*GTDR	729	723~733	1.122	0.62
LCH	P48740	MASP1_HUMAN	FGYILHTDN*R	178	170~179	1.1417	0.24
LCH	P80108	PHLD_HUMAN	N*LTTSLTESVDR	307	307~318	1.1841	0.03
LCH	P55058	PLTP_HUMAN	FYYN*ISEVK	64	61~70	1.026	0.55
LCH	P27169	PON1_HUMAN	VTQVYAEN*GTVLQGSTVASVYK	324	317~338	0.8797	0.08
LCH	O00391	QSOX1_HUMAN	N*GSGAVFPVAGADVQTLR	130	130~147	0.8605	0.09
LCH	P04278	SHBG_HUMAN	LDVDQALN*R	380	373~381	0.8799	0.11
LCH	P00734	THRB_HUMAN	GHVN*ITR	121	118~124	0.9542	0.04
LCH	P00734	THRB_HUMAN	N*FTENDLLVR	416	416~425	0.90745	0.11
LCH	P00734	THRB_HUMAN	SEGSSVN*LSPPLEQCVPDRG	205	199~218	0.9092	0.18
LCH	P04004	VTNC_HUMAN	N*ISDGFDGIPDNVDAALALPAHSY	242	242~265	0.88188	0.21
LCH	P04004	VTNC_HUMAN	NN*ATVHEQVGGPSLTSDLQAQ	86	85~105	1.06295	0.13
LCH	P04004	VTNC_HUMAN	NN*ATVHEQVGGPSLTSDLQAQSK	86	85~107	1.1733	0.06
WGA	P02675	FIBB_HUMAN	ALMDGASQLMGEN*R	394	382~395	1.067	0.10
WGA	P02675	FIBG_HUMAN	DLQSLEDILHQVEN*K	78	65~79	1.002	0.49
WGA	P01857	IGHG1_HUMAN	EEQYN*STYR	180	176~184	0.8508	0.68
WGA	P04220	MUCB_HUMAN	THTNISESHPN*ATFSAVG	217	207~224	1.1745	0.47
WGA	P04004	VTNC_HUMAN	N*ATVHEQVGGPSLTSDLQAQSK	86	86~107	1.1875	0.05
WGA	P04004	VTNC_HUMAN	NN*ATVHEQVGGPSLTSDLQA	86	85~104	1.1805	0.20
WGA	P04004	VTNC_HUMAN	NN*ATVHEQVGGPSLTSDLQAQ	86	85~105	1.181	0.11
