# Supplementary material for: An integrative strategy for quantitative analysis of the N-glycoproteome in complex biological samples
Source: Proteome Sci. 2014 Jan 15;12:4. doi: 10.1186/1477-5956-12-4 (PMC3923275; doi:10.1186/1477-5956-12-4)
Supplement: Additional file 9 — Calculation of abundance ratios of four glycopeptides between HCC patients and healthy individuals. The ratios calculated manually were similar to the ratios obtained by self-build quantitative method, which indicated the reliability of the quantitative results of our integrated research strategy. [file 1477-5956-12-4-S9.pdf]

Additional file 9: Calculation of abundance ratios of four glycopeptides between HCC patients and healthy individuals. The ratios calculated manually were similar to the ratios obtained by self-build quantitative method, which indicated the reliability of the quantitative results of our integrated research strategy. * denotes the N-glycosylation site.

Peptide sequence	Swiss-Prot	Protein Entry name	Amino acid sites	Manually calculated ratio	SD	self-build quantitative method ratio	SD
GLN*VTLSSTGR	P0C0L4	CO4A_HUMAN	1326-1336	3.013	0.193	3.015	0.070
LAN*LTQGEDQYYLR	P10909	CLUS_HUMAN	372-385	1.997	0.070	1.84	0.040
SVTWSESGQN*VTAR	P01877	IGHA2_HUMAN	38-51	0.532	0.046	0.518	0.041
FSDGLESN*SSTQFEVK	P0C0L5	CO4B_HUMAN	219-234	2.650	0.093	2.545	0.088
