# Supplementary material for: An integrative strategy for quantitative analysis of the N-glycoproteome in complex biological samples
Source: Proteome Sci. 2014 Jan 15;12:4. doi: 10.1186/1477-5956-12-4 (PMC3923275; doi:10.1186/1477-5956-12-4)
Supplement: Additional file 10 — Physiological/pathological characteristics of patients and healthy individuals enrolled in this study. [file 1477-5956-12-4-S10.pdf]

Additional file 10: Physiological/pathological characteristics of patients and healthy individuals enrolled in this study.

physiological/pathological variable	HCC patients (n=3)	Healthy individuals (n=3)
Gender		
Male	3	3
Female	0	0
Age (years)		
40-50	3	3
＞50	0	0
Weight (kg)		
51-60	0	0
61-70	2	2
71-80	1	1
Smoking history		
Yes	0	0
No	3	3
Drinking alcohol hobby		
Yes	0	0
No	3	3
Child-Pugh		
A	3	3
B	0	0
C	0	0
Tumor size (cm)		
≤2	3	0
> 2	0	0
BCLC stage		
0	3	N
A	0	N
B	0	N

The Child-Pugh system classifies the liver function from A to C, with A representing the best liver function. BCLC denotes Barcelona Clinic Liver Cancer staging system, which ranks hepatocellular carcinoma in five stages, ranging from 0 (very early stage) to D (terminal stage).
